# Exopolysaccharides From *Lactobacillus delbrueckii* subsp. *bulgaricus* OLL1073R‐1 Enhance Macrophage Bactericidal Activity Through IL‐12‐Dependent Macrophage–NK Cell Crosstalk

**DOI:** 10.1111/1348-0421.70090

**Published:** 2026-09-14

**Authors:** Chikara Kohda, Hiroki Ishikawa, Yoshihiro Kuno, Yasunari Matsuzaka, Hiroki Kono, Toshihiro Sashihara, Hirotaka Kuwata, Masayuki Iyoda

**Affiliations:** ^1^ Department of Microbiology and Immunology Showa Medical University School of Medicine Tokyo Japan; ^2^ Division of Nephrology, Department of Medicine Showa Medical University School of Medicine Tokyo Japan; ^3^ Wellness Science Labs Meiji Holdings Co., Ltd. Tokyo Hachioji Japan; ^4^ Department of Oral Microbiology and Immunology Showa Medical University School of Dentistry Tokyo Shinagawa Japan

**Keywords:** exopolysaccharides, IFN‐γ, IL‐12, *Lactobacillus delbrueckii* subsp. *bulgaricus* OLL1073R‐1, macrophage, NK cell, *Streptococcus pyogenes*

## Abstract

Exopolysaccharides (EPS) produced by *Lactobacillus delbrueckii* subsp. *bulgaricus* OLL1073R‐1 have been reported to exert immunomodulatory effects, primarily in antiviral responses; however, their role in antibacterial host defense remains unclear. Here, we investigated whether EPS enhance macrophage‐mediated killing of *Streptococcus pyogenes*, a facultative intracellular pathogen, using an in vitro co‐culture system with NK‐like cells. EPS alone did not enhance the intracellular bactericidal activity of macrophages. In contrast, EPS significantly increased bacterial killing when macrophages were co‐cultured with NK‐like cells. Mechanistically, EPS induced interleukin (IL)−12 expression in macrophages, while interferon (IFN)‐γ production was not detected when macrophages or NK‐like cells were cultured separately, but was significantly induced in the co‐culture system. Neutralization of IL‐12 or IFN‐γ significantly abrogated the EPS‐mediated enhancement of bactericidal activity, indicating that both cytokines are required for this effect. These findings support a model in which EPS stimulate macrophages to produce IL‐12, leading to NK cell activation and subsequent IFN‐γ–dependent enhancement of macrophage bactericidal function. Our results reveal a previously uncharacterized role of probiotic‐derived EPS in promoting antibacterial immunity through macrophage–NK cell crosstalk.

**Abbreviations**
EPS: exopolysaccharides
IFN: interferon
IL: interleukin
MOI: multiplicity of infection
NK cell: natural killer cell
OLL1073R‐1: *Lactobacillus delbrueckii* subsp. *bulgaricus* OLL1073R‐1
PMA: phorbol 12‐myristate 13‐acetate
RT‐qPCR: Reverse transcription‐quantitative PCR

## 1 Introduction

Exopolysaccharides (EPS) are high‐molecular‐weight extracellular polysaccharides synthesized by a wide variety of lactic acid bacteria. Based on their monosaccharide composition, EPS are generally classified into homopolysaccharides and heteropolysaccharides. Their physicochemical characteristics, including monosaccharide composition, glycosidic linkage, molecular weight, degree of branching, and three‐dimensional conformation, vary considerably among bacterial strains and determine not only their rheological properties but also their biological functions [1]. Consequently, EPS have attracted increasing attention as bioactive molecules in addition to their technological roles in improving the texture, viscosity, and stability of fermented foods.　Accumulating evidence indicates that lactic acid bacteria‐derived EPS exhibit diverse biological activities, including immunomodulatory, antioxidant, antimicrobial, antiviral, anti‐inflammatory, and intestinal barrier‐protective effects. Importantly, these biological activities are highly structure dependent. Variations in molecular architecture influence the interaction of EPS with host immune cells through pattern‐recognition receptors, including Toll‐like receptors and C‐type lectin receptors expressed on macrophages and dendritic cells, thereby regulating intracellular signaling pathways and cytokine production [2, 3]. Thus, the structure–function relationship of EPS is now recognized as a critical determinant of their immunological properties.

Among these bioactive polysaccharides, EPS produced by *Lactobacillus delbrueckii* subsp. *bulgaricus* OLL1073R‐1(OLL1073R‐1) have been reported to possess potent immunomodulatory activities that contribute to the prevention of infectious diseases. For example, EPS derived from this strain has been shown to reduce the risk of common cold and influenza [4, 5], activate natural killer (NK) cells and induce interferon (IFN)‐γ production [6, 7], and exhibit anti‐influenza virus activity, thereby reducing subsequent secondary bacterial infections [8]. However, whether OLL1073R‐1‐derived EPS enhances antibacterial host defense against bacterial pathogens has remained largely unknown.

*Streptococcus pyogenes* is an important human pathogen and has been recognized as an intracellular microorganism during severe invasive infections. It is responsible for a wide range of diseases, from mild skin and mucosal infections, such as impetigo and tonsillitis [9], to severe invasive diseases with high mortality rates, including toxic shock syndrome and necrotizing fasciitis [10, 11]. Although *S. pyogenes* is generally considered an extracellular pathogen, it can evade bacterial killing by persisting intracellularly within macrophages [12, 13] and neutrophils [14, 15]. Within macrophages, *S. pyogenes* resides in phagosomes, which contributes to its persistence during invasive infection. In these phagocytes, the M protein expressed on the bacterial surface is recognized as a key factor promoting intracellular survival [13, 15].

Previous studies demonstrated that EPS derived from OLL1073R‐1 enhances NK‐cell activity and promotes IFN‐γ production [7]. Because IL‐12 is a key cytokine that links macrophage activation to NK‐cell‐derived IFN‐γ production, we hypothesized that EPS may enhance macrophage bactericidal activity through an IL‐12–IFN‐γ‐associated pathway. To test this hypothesis, we investigated the effects of OLL1073R‐1‐derived EPS on the intracellular bactericidal activity of macrophages against *S. pyogenes* using a macrophage–NK‐like cell co‐culture system.

## 2 Materials and Methods

### 2.1 Cell Preparation

The THP‐1 human monocytic cell line (American Type Culture Collection, ATCC) was used as a source of macrophages, and the MTA cell line (Japanese Collection of Research Bioresources [JCRB] Cell Bank) was used as an NK‐like T‐cell line. Both cell lines were cultured at 37°C in a humidified atmosphere containing 5% CO_2_ in RPMI 1640 medium supplemented with 10% heat‐inactivated fetal bovine serum (FBS; NICHIREI Biosciences Inc., Japan) and 1% penicillin/streptomycin (FUJIFILM Wako Pure Chemical, Japan). Cells were seeded in 24‐well plates at 5 × 10^6^ cells/well. THP‐1 cells (2 × 10^6^ cells/mL) were differentiated into macrophages by treatment with 200 nM phorbol 12‐myristate 13‐acetate (PMA; Sigma‐Aldrich).

### 2.2 Bacterial Strain and Infection Protocol

*S. pyogenes* ATCC 10389 was used in this study. Bacteria were cultured in brain heart infusion (BHI; BD‐Difco) broth at 37°C for 16 h, collected by centrifugation, and stored at −70°C until use. Macrophages were stimulated with EPS (0, 5, 50, 100, or 200 μg/mL) for 20 h before the binding and killing assays. The culture medium was then replaced with antibiotic‐free medium, and cells were infected with *S. pyogenes* at a multiplicity of infection (MOI) of 50 bacteria per cell. OLL1073R‐1 was obtained from Meiji Co. Ltd. (Japan).

### 2.3 Preparation of EPS

EPS was prepared as previously described [7]. Briefly, 10% (w/w) skim milk fermented with OLL1073R‐1 was mixed with 10% (w/v) trichloroacetic acid and centrifuged to obtain the supernatant. The supernatant was mixed with two volumes of ethanol, and the precipitate was collected. The precipitate was dissolved in water and dialyzed using a membrane with a molecular weight cutoff of 6000–8000 Da (Repligen, USA). The dialyzed solution was incubated at 37°C with DNase and RNase (Sigma‐Aldrich), each at a final concentration of 1 μg/mL, for 6 h, followed by incubation with proteinase K (0.1 mg/mL; Sigma‐Aldrich) for 18 h. After enzyme inactivation, the solution was again precipitated with ethanol, and the final product was dialyzed and lyophilized.

### 2.4 Co‐Culture System

THP‐1 cells were plated at 2.5 × 10^6^ per well in a 24‐well flat‐bottomed tissue culture plate and stimulated with 100 μL of various concentrations of EPS for 20 h. After 20 h, NK cells　(2.5 × 10^6^ per well) were added and the culture was incubated for an additional 24 h. After 24 h, the NK cells were centrifuged and collected, and the culture supernatant was removed. The NK cells, resuspended in a medium containing EPS but no antibiotics, were added to THP‐1 cells once again; both cell lines were then used in a bacterial infection assay.

### 2.5 Binding Assay

After infection, plates were centrifuged at 450 × g for 5 min to promote bacterial attachment and incubated at 4°C for 60 min. Extracellular bacteria were removed by washing three times with phosphate‐buffered saline (PBS). Macrophages were lysed with 0.1% Triton X‐100, and viable bacteria were quantified by serial dilution and plating on BHI agar.

### 2.6 Intracellular Killing Assay

After infection, plates were centrifuged at 450 × g for 5 min and incubated at 37°C for 60 min to allow bacterial uptake. Extracellular bacteria were removed by washing with PBS, and gentamicin (10 μg/mL) was added to eliminate remaining extracellular bacteria. After 1 h of gentamicin treatment (defined as time 0), intracellular viable bacteria were quantified. After an additional 6 h, intracellular bacterial counts were determined again. The reduction in bacterial counts was defined as macrophage intracellular killing activity. For cytokine neutralization experiments, anti–IL‐12 p35 monoclonal antibody and anti–IFN‐γ monoclonal antibody (Proteintech, USA) were used. Corresponding non‐neutralizing control antibodies (NeutraControl IL‐12 p35 and NeutraControl IFN‐γ; Proteintech) served as negative controls.

### 2.7 Analysis of IL‐12 and IFN‐γ mRNA Expression

Reverse transcription‐quantitative PCR (RT‐qPCR) was performed to assess IL‐12 and IFN‐γ mRNA expression in macrophages and NK cells 24 h after EPS stimulation. Total RNA was extracted using the RNeasy Mini Kit (Qiagen, Japan), including on‐column DNase treatment to remove genomic DNA contamination. cDNA was synthesized using the QuantiTect Reverse Transcription Kit (Qiagen), including no‐RT controls. Primers and probes were used with the TaqMan Gene Expression Assay (Thermo Fisher Scientific Inc, Japan), and amplification was performed on a QuantStudio 3 Real‐Time PCR System (Thermo Fisher Scientific Inc) according to the manufacturer's instructions. Experiments were conducted with 3–6 biological replicates.

### 2.8 Measurement of IFN‐γ Induced By EPS

THP‐1 cells or/and NK cells were plated at 2.5 × 10^6^ per well in a 48‐well flat‐bottomed tissue culture plate and stimulated with 100 μL of EPS (200, 50, and 0 μg/mL) for 24 h. In the co‐culture system, THP‐1 cells (2.5 × 10^6^ per well) were first treated with EPS for 20 h and then added with NK cells (2.5 × 10^6^ per well) for 24 h. The culture supernatant was collected, and the level of IFN‐γ was measured by BD Opt EIA Human IFN‐γ ELISA set (BD Biosciences).

### 2.9 Statistical Analysis

All intracellular growth assays were independently repeated at least three times. Data are presented as the mean ± standard deviation (SD) of triplicate determinations. Student's *t*‐test and one‐way ANOVA were used for statistical comparisons. A *p* value < 0.01 was considered statistically significant.

## 3 Results

### 3.1 Effect of EPS on Intracellular Survival of *S. pyogenes* in Macrophages

The results of the binding assay are shown in Figure 1A. Treatment of macrophages with EPS (0, 5, 50, 100, or 200 μg/mL) did not affect bacterial attachment compared with IFN‐γ– (10 units/well) or LPS‐treated (100 ng/mL) macrophages. We next assessed intracellular killing activity (Figure 1B). Macrophages treated with EPS alone showed no significant increase in intracellular bactericidal activity compared with untreated controls (EPS = 0 μg/mL). Although a decreasing trend in intracellular bacterial counts was observed 6 h after infection in EPS‐treated macrophages, the reduction was not statistically significant. In contrast, IFN‐γ– and LPS‐treated macrophages showed a significant reduction in intracellular bacteria. These results indicate that EPS alone does not enhance macrophage intracellular bactericidal activity against *S. pyogenes*.

**Figure 1:**
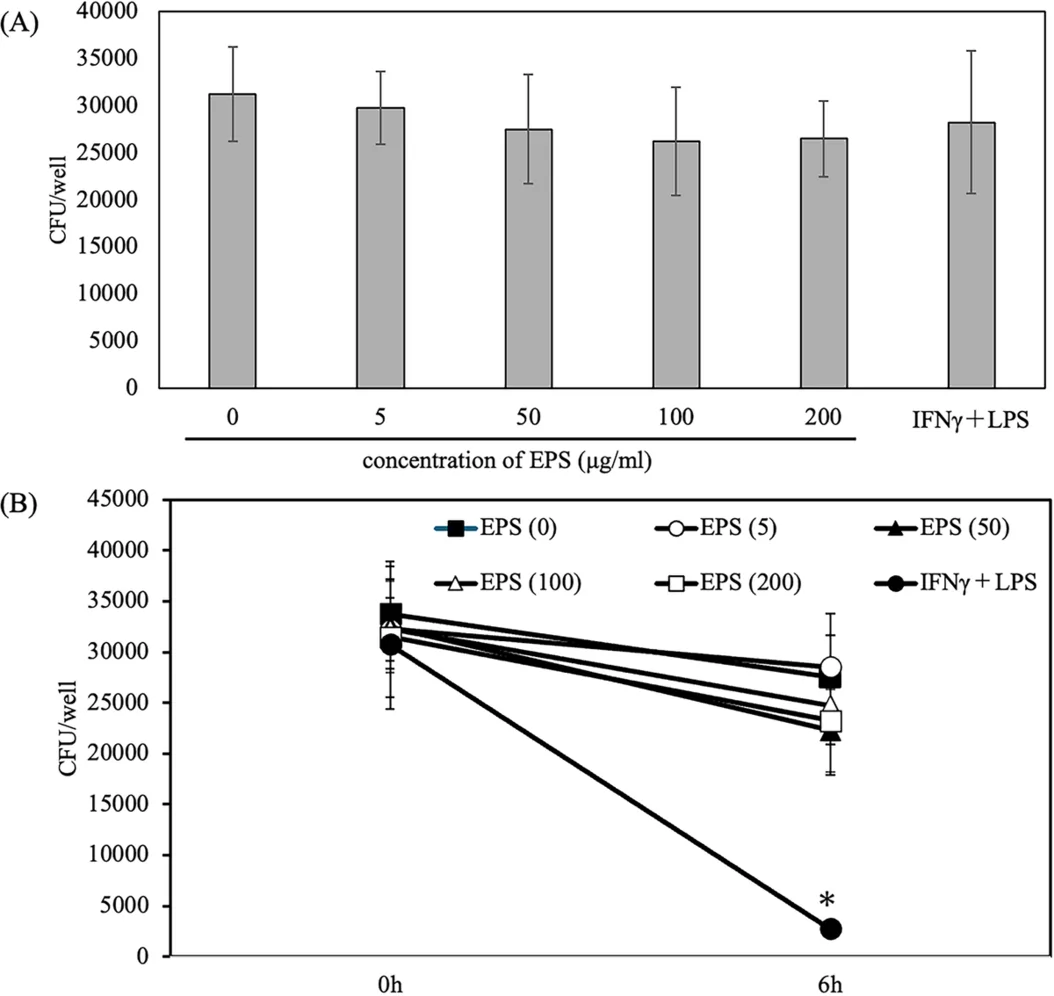
EPS alone does not enhance macrophage intracellular killing of *S. pyogenes*. Macrophages were seeded in 24‐well plates and stimulated with EPS (0–200 μg/mL) for 20 h prior to infection. Cells were infected with *S. pyogenes* at a MOI of 50:1. (A) Binding assay. Plates were incubated at 4°C for 60 min to allow bacterial attachment without internalization. After washing to remove unbound bacteria, cell‐associated bacteria were quantified. (B) Intracellular killing assay. Following phagocytosis at 37°C for 60 min, extracellular bacteria were removed by washing and gentamicin treatment (10 μg/mL, 1 h). Intracellular viable bacteria were quantified immediately after gentamicin treatment (0 h) and after an additional 6 h of incubation. Data are presented as mean ± standard deviation (SD) of three independent experiments, each performed in triplicate. Statistical significance was determined by one‐way ANOVA. *p < 0.01 versus EPS (0 μg/mL).

### 3.2 Effect of Co‐Culture with NK Cells on EPS‐Induced Intracellular Bactericidal Activity in Macrophages

Macrophages were then co‐cultured with NK cells, and intracellular bactericidal activity was evaluated (Figure 2). In the presence of EPS (200 and 100 μg/mL), macrophages exhibited enhanced intracellular bactericidal activity comparable to that observed in IFN‐γ– and LPS‐treated cells. In contrast, when stimulated with low concentrations of EPS (50, 5, and 0 μg/mL), intracellular bactericidal activity did not increase even in the co‐culture system.

**Figure 2:**
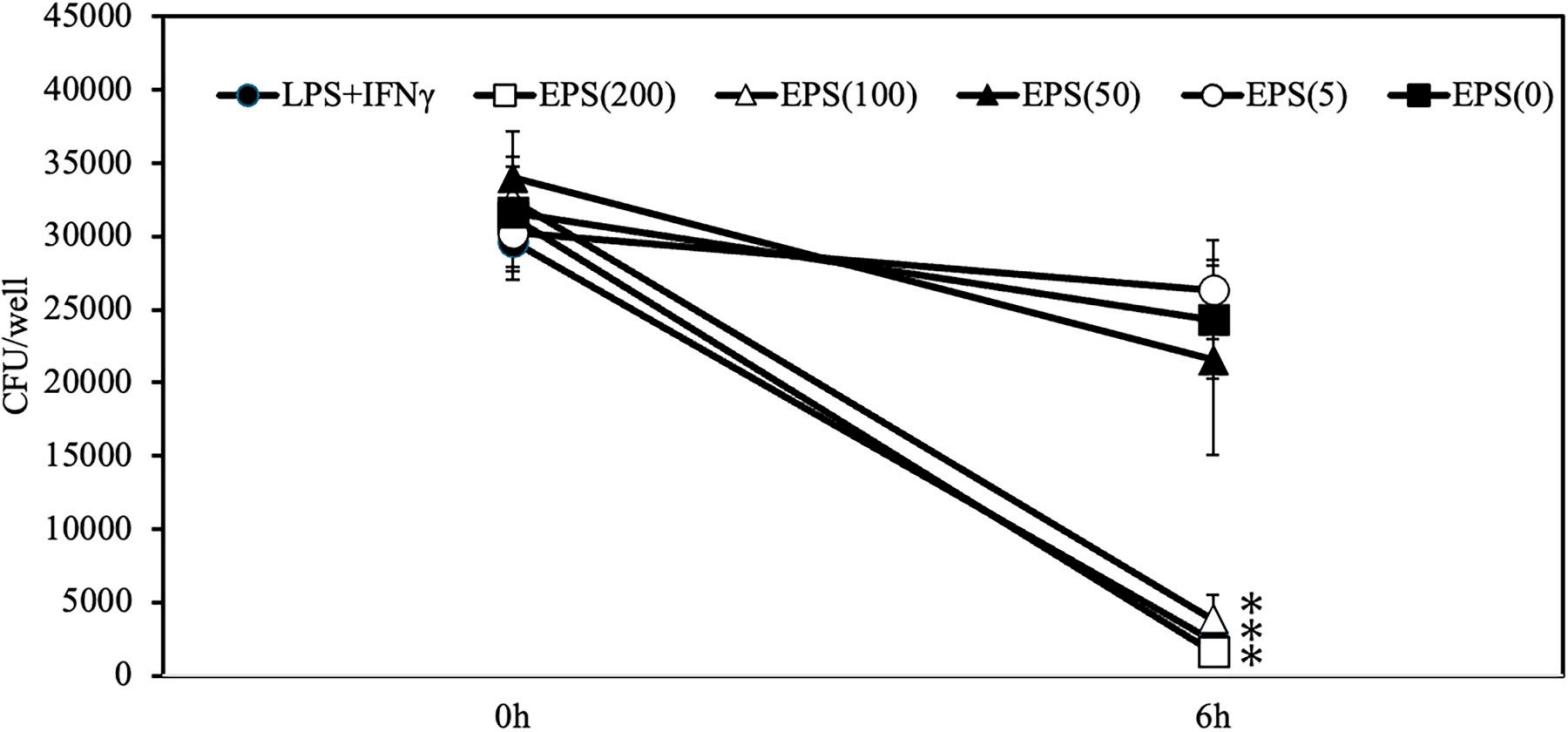
NK‐like cells are required for EPS‐mediated enhancement of macrophage bactericidal activity. Macrophages and NK‐like MTA cells were co‐cultured in 24‐well plates and stimulated with EPS (0–200 μg/mL) for 20 h. Cells were infected with *S. pyogenes* (MOI = 50:1), and intracellular killing activity was assessed. Data are presented as mean ± SD of three independent experiments, each performed in triplicate. Statistical significance was determined by one‐way ANOVA. *p < 0.01 versus EPS (0 μg/mL).

### 3.3 EPS‐Induced Cytokine Gene Expression in Macrophages and NK Cells

Next, we investigated the mechanism by which the intracellular killing activity of macrophages increases when co‐cultured with NK cells. We evaluated the cytokine induction activity of EPS on IL‐12 and IFN‐γ, which are involved in the activation of macrophages and NK cells, using RT‐qPCR.

In macrophages stimulated with EPS (200 and 100 μg/mL), IL‐12 mRNA　expression was significantly upregulated, whereas IFN‐γ mRNA expression was not detected (Figure 3A). In addition, IL‐12 mRNA expression was concentration‐dependent with respect to EPS (Figure 3A). In contrast, stimulation of NK cells with EPS did not induce the expression of either IL‐12 or IFN‐γ mRNA (Figure 3B). These findings indicate that EPS directly induces IL‐12 expression in macrophages but does not directly stimulate NK cells to produce IFN‐γ.

**Figure 3:**
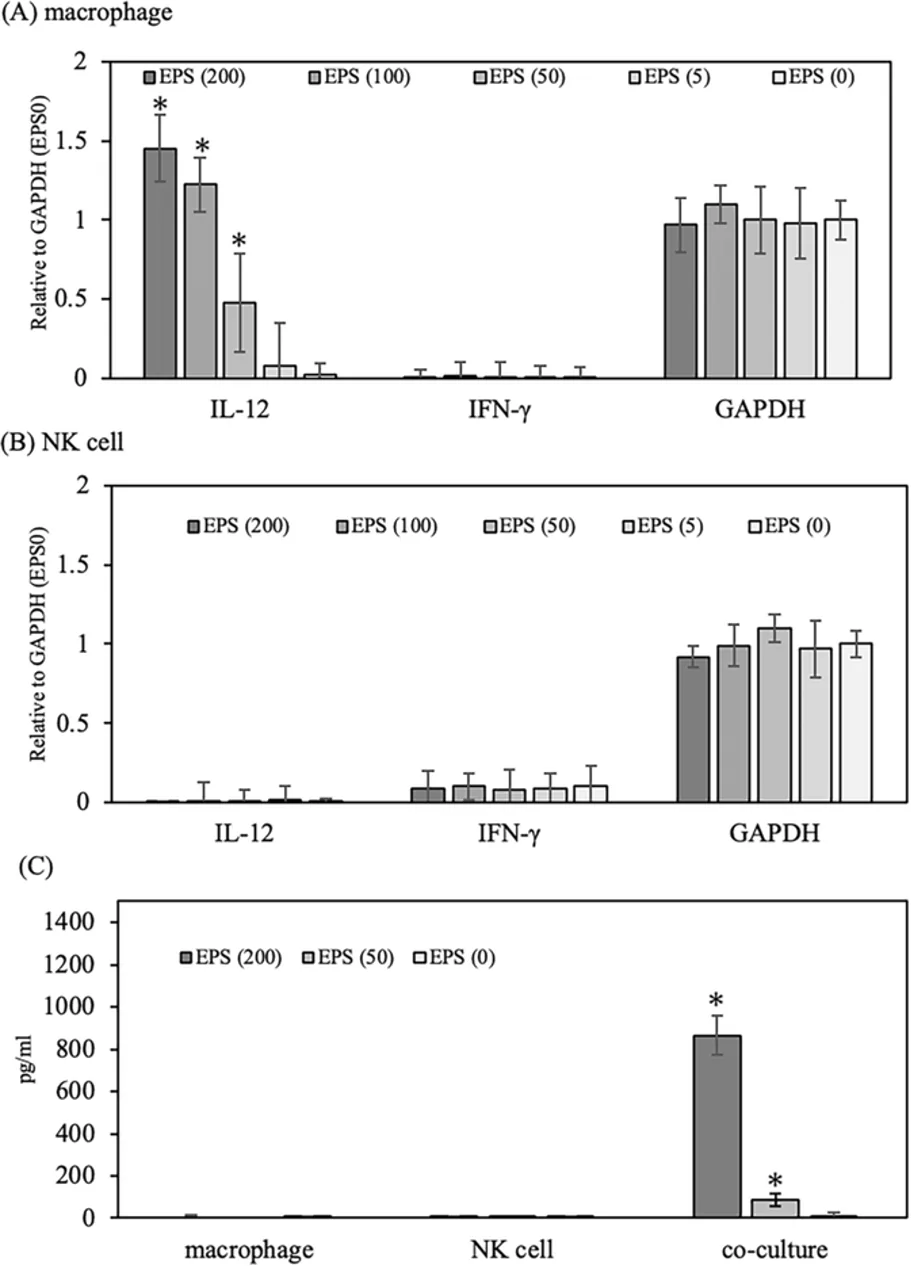
EPS induces IL‐12 expression in macrophages and IFN‐γ production only in a macrophage–NK co‐culture system. (A) IL‐12 and IFN‐γ mRNA expression in macrophages. (B) IL‐12 and IFN‐γ mRNA expression in NK‐like cells. Relative mRNA expression is shown. (C) IFN‐γ production measured by ELISA. Data represent mean ± SD of 3–6 independent biological replicates. Statistical significance was determined by Student's *t*‐test. *p < 0.01 versus EPS (0 μg/mL).

### 3.4 Production of IFN‐γ Induced By EPS Stimulation in a Co‐Culture System

To test our hypothesis that “EPS may enhance the bactericidal activity of macrophages via a pathway involving IL‐12 and IFN‐γ,” we measured IFN‐γ production in a co‐culture system using the ELISA method. When macrophages and NK cells were stimulated with EPS individually, no IFN‐γ production was observed. In the co‐culture system, no IFN‐γ production was observed when cells were not stimulated with EPS, whereas significant, concentration‐dependent IFN‐γ production was observed when cells were stimulated with EPS (Figure 3C). It was suggested that the lack of increased intracellular bactericidal activity against *S. pyogenes* in response to EPS stimulation at concentrations of 50 μg/mL or less (Figure 2) was due to the fact that insufficient IFN‐γ was produced to activate the intracellular bactericidal capacity of macrophages.

### 3.5 Effect of Cytokine Neutralization on EPS‐Induced Intracellular Bactericidal Activity

To clarify the role of cytokines in EPS‐induced intracellular bactericidal activity, neutralizing antibodies against IL‐12 and IFN‐γ were added to the macrophage–NK cell co‐culture system. Cells were stimulated with 200 μg/mL EPS in the presence of cytokine‐specific neutralizing antibodies (2 μg/mL) for 24 h, and intracellular bactericidal activity was assessed by quantifying intracellular bacterial counts. No effect of neutralizing antibody treatment was observed on the number of streptococci infecting macrophages at 0 h (Figure 4B).　Addition of anti–IL‐12 or anti–IFN‐γ antibodies significantly reduced EPS‐induced intracellular bactericidal activity. In contrast, non‐neutralizing control antibodies had no effect (Figure 4C). These results demonstrate that both IL‐12 and IFN‐γ are required for EPS‐mediated enhancement of macrophage intracellular bactericidal activity.

**Figure 4:**
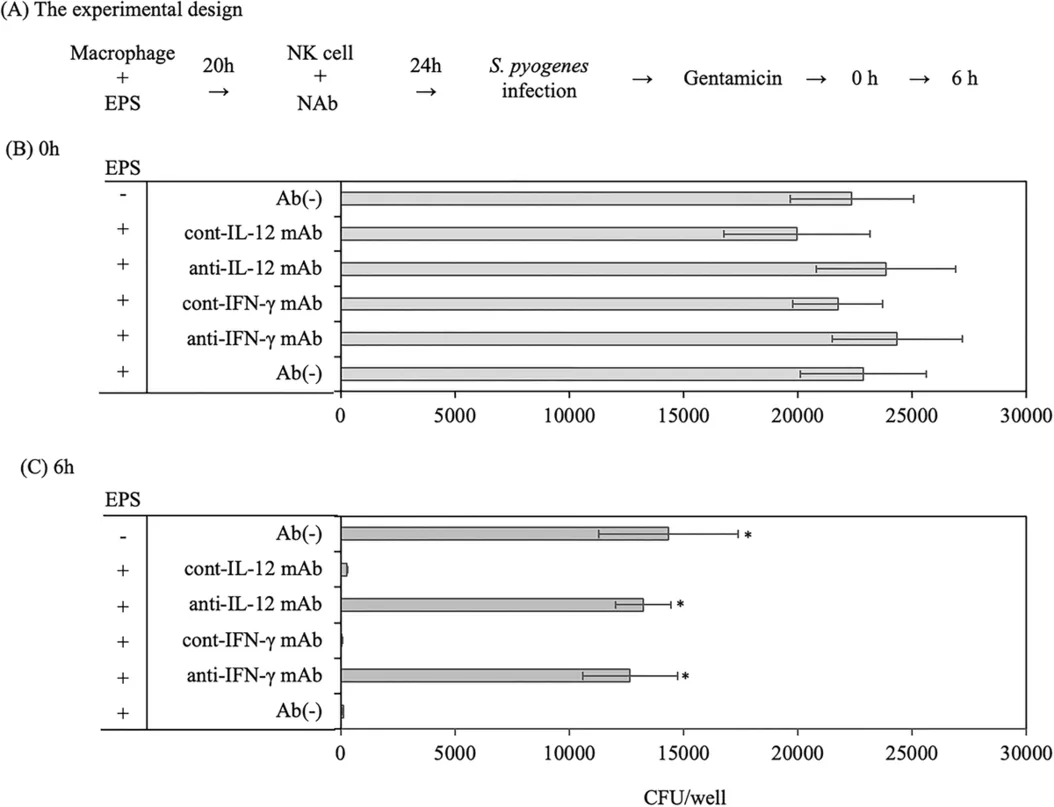
Neutralization of IL‐12 or IFN‐γ abolishes EPS‐mediated enhancement of macrophage bactericidal activity. (A) The experimental design for cytokine neutralization experiments. (B) Intracellular bacterial counts at 0 h. (C) Intracellular bacterial survival 6 h after infection in the presence or absence of neutralizing antibodies. Data are presented as mean ± SD of three independent experiments, each performed in triplicate. Statistical significance was determined by one‐way ANOVA. *p < 0.01 versus EPS‐treated group without antibody.

## 4 Discussion

The present study provides the first evidence that EPS derived from *Lactobacillus delbrueckii*subsp. *bulgaricus* OLL1073R‐1 enhances the intracellular bactericidal activity of macrophages against *Streptococcus pyogenes* through macrophage–NK cell crosstalk. EPS alone did not enhance macrophage bactericidal activity; however, a marked increase was observed in the presence of NK‐like cells, indicating that EPS enhances antibacterial host defense indirectly through immune cell interactions rather than by directly activating macrophages.

Mechanistically, our findings support a model in which EPS stimulate macrophages to produce IL‐12, leading to NK‐cell activation and subsequent IFN‐γ production that enhances macrophage antimicrobial activity [16, 17]. This model is supported by three observations: (i) EPS induced IL‐12 expression in macrophages; (ii) IFN‐γ production was detected only in the macrophage–NK cell co‐culture, but not in either cell type cultured alone; and (iii) neutralization of IL‐12 or IFN‐γ abolished the EPS‐mediated enhancement of intracellular bactericidal activity. Although the present study focused on IL‐12 and IFN‐γ because they constitute a well‐established macrophage–NK cell signaling pathway, the involvement of additional cytokines, such as IL‐18 and TNF‐α, cannot be excluded and should be addressed in future studies.

Previous studies using murine splenocyte cultures demonstrated that OLL1073R‐1‐derived EPS enhances NK‐cell activity and IFN‐γ production [6, 7]. At first glance, these findings appear inconsistent with our observation that EPS did not induce detectable IFN‐γ production in NK‐like cells cultured alone. However, this apparent discrepancy is most likely explained by differences in the experimental systems. Splenocyte cultures contain multiple accessory immune cells, including macrophages and dendritic cells, which produce IL‐12 and other cytokines required for optimal NK‐cell activation. In contrast, our simplified co‐culture system enabled us to directly evaluate macrophage–NK cell interactions and to distinguish the direct effects of EPS on NK‐like cells from the indirect effects mediated by macrophage‐derived IL‐12. Such mechanistic discrimination could not be achieved using whole splenocyte cultures. Consistent with this interpretation, IFN‐γ production was significantly increased only in the macrophage–NK cell co‐culture, supporting a critical role for macrophage‐derived IL‐12 in EPS‐mediated NK‐cell activation. Thus, rather than contradicting previous reports, the present study extends them by identifying macrophage‐derived IL‐12 as a key mediator linking EPS stimulation to NK‐cell activation and subsequent IFN‐γ production.

The immunomodulatory effects of OLL1073R‐1‐derived EPS have previously been investigated primarily in the context of antiviral immunity [18, 19]. Our findings broaden this concept by demonstrating that these EPS also enhance host defense against a bacterial pathogen with an intracellular phase. Because *S. pyogenes* can survive within phagocytic cells and evade immune clearance, enhancement of macrophage intracellular killing may represent an important mechanism by which probiotic‐derived EPS contribute to antibacterial immunity. Collectively, these findings extend the biological functions of probiotic‐derived EPS beyond antiviral immunity and identify macrophage–NK cell crosstalk as a previously unrecognized mechanism underlying EPS‐mediated antibacterial host defense.

Several limitations should be acknowledged. First, the present study employed an in vitro cell line‐based model, and therefore, the physiological relevance of these findings requires validation in vivo. Second, although IFN‐γ protein production was confirmed by ELISA, IL‐12 induction was assessed only at the mRNA level, and future studies should evaluate IL‐12 protein production and downstream signaling. Nevertheless, the functional importance of IL‐12 is supported by the observation that neutralization of IL‐12 abolished the EPS‐mediated enhancement of intracellular bactericidal activity, suggesting that biologically active IL‐12 was produced in the co‐culture system. Third, the contribution of other cytokines, including IL‐18 and TNF‐α, was not examined. Furthermore, the molecular mechanisms by which EPS are recognized by macrophages and the structural features responsible for their immunomodulatory activity remain unclear. Elucidating the receptors and structure–function relationships underlying EPS‐mediated immune activation will provide important insights into their biological functions.

In conclusion, OLL1073R‐1‐derived EPS enhances macrophage intracellular bactericidal activity against *S. pyogenes*through an IL‐12–NK cell–IFN‐γ‐dependent mechanism. These findings reveal a previously unrecognized role of probiotic‐derived EPS in antibacterial immunity and provide a mechanistic basis for the development of EPS‐based immunomodulatory strategies against intracellular bacterial infections.

## Author Contributions

**Chikara Kohda:** investigation, writing–original draft. **Hiroki Ishikawa:** data curation, investigation. **Yoshihiro Kuno:** data curation, investigation. **Yasunari Matsuzaka:** data curation, investigation. **Hiroki Kono:** data curation, funding acquisition, supervision, writing – review and editing. **Toshihiro Sashihara:** project administration, funding acquisition, supervision, writing – review and editing. **Hirotaka Kuwata:** data curation, supervision, writing–review and editing. **Masayuki Iyoda:** funding acquisition, project administration, supervision, writing – review and editing.

## Ethics Statement

The authors have nothing to report.

## Conflicts of Interest

Kono H. and Sashihara T. are employees of Meiji Holdings Co. Ltd. This research was funded by a joint research effort conducted by the author and Meiji Holdings Co. Ltd.

## Data Availability

The data that support the findings of this study are available on request from the corresponding author. The data are not publicly available due to privacy or ethical restrictions. The data that support the findings of this study are available within the article.
